# Metabolic syndrome in HIV-infected individuals: underlying mechanisms and epidemiological aspects

**DOI:** 10.1186/1742-6405-10-32

**Published:** 2013-12-13

**Authors:** Adelzon A Paula, Melissa CN Falcão, Antonio G Pacheco

**Affiliations:** 1 Programa de Computação Científica, Fundação Oswaldo Cruz, Rio de Janeiro, Brasil

**Keywords:** HIV, Metabolic syndrome, Cardiovascular risk, AIDS, HAART

## Abstract

The success of highly active antiretroviral therapy (HAART) has determined a dramatic decline in AIDS- and immunodeficiency-related causes of death in the HIV-infected population. As life-expectancy increases, such individuals have become gradually exposed not only to the effects of aging itself, but also to the influence of environmental risk factors, which are known to act in the general population. These features can lead to obesity, diabetes mellitus and ultimately cardiovascular diseases (CVD). Metabolic complications and abnormal fat distribution were frequently observed after a few years of antiretroviral therapy and, as the array of antiretroviral drugs became broader, long term metabolic alterations are becoming far more common worldwide. Nevertheless, the risk of not being on HAART is overwhelmingly greater than the metabolic adverse events in terms of morbidity and mortality events. HIV/HAART-induced metabolic unbalances overlap in some extent the components of Metabolic Syndrome (MetS) and its high rates in the HIV population place infected individuals in an elevated CVD risk category. MetS can explain at least in part the emergence of CVD as the major morbidity and mortality conditions in the HIV population. In this review we convey information on the underlying aspects of MetS during HIV infection, highlighting some physiopathological and epidemiological features of this comorbidity along with the role played by HIV itself and the synergy action of some antiretroviral drugs. Considerations on MetS management in the HIV population are also depicted.

## Review

### Introduction

The introduction and widespread use of highly active antiretroviral therapy (HAART) in the mid 1990’s, has led HIV-infected individuals to experience a dramatic decline in immunodeficiency-related events, including causes of death [1-3]. As a consequence, life-expectancy increased, which exposed them to the effects of aging itself, including the influence of the same environmental risk factors known to act in the general population and contributing to the occurrence of obesity, diabetes mellitus (DM), and cardiovascular diseases (CVD) [4,5].

Metabolic syndrome (MetS) has been playing a major role as a marker for metabolic disorders [6]. According to the Third National Health and Nutrition Examination Survey, prevalence of MetS in the general US population has been estimated in 25% and this number has been growing continuously over time [7]. A recent British study on a cohort of middle-aged men reported the prevalence of MetS at 26% [8]. The increasing prevalence of MetS relates to the growing tendencies verified for its underlying causes, such as unbalanced food intake, physical inactivity and obesity, which peaked in the developed world and have been suggested to be independent risk factors for the development of the syndrome [8].

MetS encompasses a cluster of risk factors leading to CVD as primary clinical outcome and contribute to higher risks of DM. Such factors include obesity (mainly central adiposity), defective glucose metabolism (DM, impaired glucose tolerance, or impaired fasting glycaemia), raised blood pressure, and elevated TG and low HDL-c levels. Notwithstanding the cardiovascular outcomes, individuals with MetS are thought to be more susceptible to a range of conditions including some cancers [9], polycystic ovary syndrome [10] and asthma [11].

Since its first comprehensive recognition in 1988, when Reaven described the relation between insulin resistance, plasma insulin levels, glucose intolerance and hypertension [12], MetS has been subject to a number of operational definitions. Despite the absence of a consensual definition to date, which dampers accurate prevalence estimates, the different working definitions for the syndrome converge to some extent. Four of the main MetS operational definitions and their differential and overlapping components can be seen in Table 1.

**Table 1:** Work definitions for MetS

**Definition**	**EGIR (1999)**[13]	**NCEP/ATP III (2001)**[14]	**AHA-NHLBI (2005)**[15]	**IDF (2006)**[16]
**Mandatory criteria**	Insulin resistance defined as the top 25% of the fasting insulin values among nondiabetic individuals	None	None	Waist circumference^#^ with ethnicity-specific values
**Aditional criteria**	At least two of the following:	At least three of the following:	At least three of the following:	At least two of the following:
**Central obesity**	Waist circumference ≥ 94 cm (male), ≥ 80 cm (female)	Waist circumference ≥ 102 cm or 40 inches (male), ≥ 88 cm or 35 inches (female)	Waist circumference ≥ 102 cm or 40 inches (male), ≥ 88 cm or 35 inches (female)	See mandatory criteria
**Dyslipidemia**	TG ≥ 2.0 mmol/L and/or HDL-C < 1.0 mmol/L or treated for dyslipidemia	TG ≥ 1.7 mmol/L (150 mg/dl) OR HDL-C < 40 mg/dL (male), < 50 mg/dL (female)	TG ≥ 1.7 mmol/L (150 mg/dl) OR HDL-C < 40 mg/dL (male), < 50 mg/dL (female)	TG > 150 mg/dL (1.7 mmol/L), or specific treatment for this lipid abnormality OR HDL-C < 40 mg/dL (1.03 mmol/L) in males, < 50 mg/dL (1.29 mmol/L) in females, or specific treatment for this lipid abnormality
**Blood pressure**	≥ 140/90 mmHg or antihypertensive medication	≥ 130/85 mmHg or antihypertensive medication	≥130/85 mmHg	Systolic BP > 130 OR diastolic BP >85 mmHg, or treatment of previously diagnosed hypertension
**Glucose metabolism**	Fasting plasma glucose ≥ 6.1 mmol/L	Fasting plasma glucose ≥ 6.1 mmol/L (110 mg/dl)	Fasting plasma Glucose ≥ 100 mg/dL	Raised fasting plasma glucose: >100 mg/dL (5.6 mmol/L), or previously diagnosed tDM

^#^If BMI is >30 kg/m^2^, central obesity can be assumed and waist circumferencedoes not need to be measured; BP: Blood Pressure.

### MetS in HIV infection

Despite the unquestionable success of HAART, prevalence of DM, insulin resistance, blood pressure fat redistribution and mainly dyslipidemia have substantially increased after its global scaling up [17]. Though the actual numbers of MetS in HIV populations are still debatable, reported prevalences for MetS in the HIV population can be regarded as high, ranging from 11.2% up to 45.4% (Table 2). The high rates of MetS in the HIV-infected population place it in a CVD high-risk category, turning MetS into a major public health concern [18-20].

**Table 2:** MetS prevalences among different HIV populations

**MetS criteria**	**Prevalence (%)**	**Remarks**	**Reference/year**
**NCEP-ATP III**	45.4	n = 553, Italy.	[21]/2002
		On HAART	
	17	n = 710, Spain.	[22]/2005
	22	n = 1243 Italy.	[23]/2006
		SIMONE multicenter study	
	15.8	n = 146, Spain Madrid.	[24]/2006
		On HAART, plus 159 HIV negative patients matched by BMI	
	18	n = 788.	[25]/2007
		Lipodystrophy case definition cohort (international multicenter)	
	25.5	n = 471, USA.	[26]/2007
		NHANES, matched for age, gender, ethnicity, tobacco use	
	20.8	n = 1243, Italy.	[25]/2007
		SIMONE multicenter study	
**EGIR**	39.8	n = 201, Italy.	[27]/2002
		On HAART.	
	33.1	n = 287, Italy.	[28]/2003
		On HAART	
**NHLBI**	24	n = 77, USA.	[20]/2006
		NFHL Study	
**IDF**	14	n = 788.	[25]/2007
		Lipodystrophy case definition cohort (international multicenter)	
	11.4	n = 210, Spain.	[29]/2007

The natural course of HIV infection is associated with particular unbalances in lipid levels. The dynamics of HIV infection determine an initial decrease in HDL-c followed by a decrease in LDL-c levels. In more advanced stages, there is an increase in TG and in VLDL-c levels with a strong correlation between serum IFN-α levels and TG clearance time [30]. Notwithstanding, there is evidence for different MetS pathways among HIV individuals under HAART treatment, since a recent report indicated an unbalanced relationship between HDL-c and TG in the presence of high TG levels or another component of MetS [31], which claims for an appropriate MetS definition in HIV infection.

### Physiopathology of MetS in HIV-infected individuals

Since the description of abnormal fat distribution following a few years on HAART with Protease Inhibitors (PI), chiefly ritonavir plus saquinavir combination [32], metabolic changes in HIV individuals have been widely studied. The main features included dyslipidemia, insulin resistance, and lipodystrophy. Many of these phenotypic and metabolic changes fit MetS criteria [18] and therefore, there is growing concern that metabolic complications associated to HIV and HAART may lead to increased risk for cardiovascular events. Such reasoning can explain, at least partially, the emergence of CVD as causes of morbidity and death in the HIV population.

HIV infection is associated with deregulated inflammatory response, through suppressing genes necessary to extinguish inflammation. In such context, HIV-infected monocytic cells have downregulated expression of the tyrosine kinase RON, a negative regulator of the inflammatory process and HIV transcription as well, via ubiquitin-proteosome degradation [33]. This long term inflammatory environment along with higher white blood cells count act as a metabolic risk factor in the pathogenesis of HIV [34].

While obesity is a central component of MetS, adipose tissue is a dynamic source of several proinflammatory cytokines, chemokines, growth factors and complement proteins, which can alter endothelial cells integrity and contribute to the atherosclerosic process [35]. This constitutive low-grade inflammatory status is characterized by increased plasma levels of TNF-α and IL-6 and other mediators of inflammation [36]. The interplay between HIV-triggered low-grade inflammatory injury, inbalances in lipid and glucose metabolism, and fat redistribution has already been described, with soluble urokinase plasminogen activator receptor (suPAR) emerging as a stronger predictor of dysmetabolism than TNF-α and IL-6 [37].

Insulin resistance is thought to determine excessive adipokine production yielding to endothelial dysfunction. As it progresses towards MetS and DM, the ongoing process of endothelial damage, along with inflammation, thrombosis and oxidation orchestrate at the vessel wall to produce atherosclerotic plaques [36]. Accordingly, caloric restriction-induced weight loss contributes to the regulation of a wide variety of inflammation-related molecules adipose tissue and upregulated the expression of molecules with anti-inflammatory properties [38].

### HIV-related risk factors

Inflammation is thought to be a major determinant in the pathogenesis of both DM and atherosclerosis. However, the key inflammatory molecules involved in atheroma and DM in HIV individuals on HAART are poorly understood [39]. Epicardial and thoracic periaortic fat deposition have been associated to high levels of hsCRP, insulin resistance and subclinical atherosclerosis in virologically suppressed HIV-infected patients on HAART and both have been related to MetS [40,41]. In fact, epicardial fat storage and some lipodystrophy phenotypes and well established risk factors for atherosclerosis seem to be associated [42].

A case–control study performed among HIV-infected ART-naïve Africans showed a high prevalence of MetS and increased arterial stiffness, considered an early marker of atherosclerosis. In this report, prevalence of impaired fasting glucose and DM, levels of fasting TG and the atherogenic dyslipidemia ratio were higher in HIV-individuals than in controls. Elevated blood pressure prevalence was high but comparable in both groups [43].

Although to a lesser degree than HAART, HIV infection act as an independent risk factor for atherosclerosis development and cardiovascular damage, been responsible for the increased prevalence of MetS and arterial function impairment [44]. HIV-specific mechanisms include immune dysfunction and increased inflammatory response leading to increased thrombosis and changes in lipid levels and cholesterol metabolism, which are also responsible for MetS and cardiovascular risk in the general population. Tat, a key molecule in HIV replication and pathogenesis can affect both mesenchymal stem cells survival and differentiation by downregulating the expression of VEGF-induced endothelial markers and this might play an instrumental role in vessel damage and in the atherosclerotic lesions observed in HIV infection [45].

The pathogenesis of dyslipidemia in HIV-infected individuals has been associated with increased apolipoprotein levels, increased hepatic synthesis of VLDL-c, decreased clearance of TG [46] and also to the effects of viral infection itself, acute-phase proteins and increase in circulating cytokines such as IL-6 and IFN-α [47]. In fact, lipid unbalances are common in art-naïve HIV-infected individuals even in the absence of major host-related risk factors for dyslipidemia, such as high blood pressure, DM and obesity [44].

HIV-1 infection *itself* is able to elicit adipose tissue alterations critical to lipodystrophy causation through adipose tissue gene expression alterations. Subcutaneous adipose tissue from infected individuals bears reduced mRNA levels of cytochrome c oxidase subunit II compared to non-infected individual. These concentrations decreased further in association with HAART [48].

### Antiretroviral-related risk factors

HAART therapy has both positive and deleterious effects on cardiovascular risk. Cumulative evidence has pointed to the relation between different metabolic disorders and HAART use, including insulin resistance, hyperlipidaemia, and lipodystrophy [49], even though it remains controversial whether these effects can be directly ascribed to antiretroviral drugs [26]. Antiretroviral-driven suppression of HIV replication seem to act as double-edged sword since it can reduce and also increase HIV-related cardiovascular risk through its toxicity [50].

Despite effective treatment with HAART, some degree of chronic immune activation may persist. In the SMART trial, participants bearing ≤400 copies/mL of HIV RNA also had elevated hsCRP and IL-6 levels in 38% and 60%, respectively, in comparison to normal individuals form cohorts for cardiovascular outcomes [51]. HIV-infected individuals have higher blood levels of major inflammation markers such as IL-6, hsCRP and p-selectin, considered independently associated with increased cardiovascular risk [52]. Interrupting ART use may further increase the risk of death by raising IL-6 and D-dimer levels [53].

HAART toxicity depends on the antiretroviral drug used and may include adverse lipoprotein changes, insulin resistance, inflammation, platelet dysfunction, and vascular injury. Studies performed *in vitro* have demonstrated that some HAART regimens, such as those including zidovudine, some NNRTI (e.g. efavirenz) and indinavir induce toxicity through induction of cardiomyocyte and endothelial cell apoptosis leading to endothelial dysfunction and vascular damage [54]. Thus, compared to untreated HIV infection, the net effect of starting antiretroviral therapy on cardiovascular disease risk is unknown as it may increase or decrease the overall risk [55]. Studies suggest that conventional risk factors will play major role in the development of CVD in HIV patients, as seen in the general population and such risk factors urge to be targeted by prevention strategies [56,57].

The unbalances in glucose metabolism depend on the particular antiretroviral drug in use. Treatments with stavudine [58], zidovudine [59,60], lamivudine [59] or didanosine [58], as well as indinavir [61,62], or lopinavir/ritonavir [60], and efavirenz have [63,64] been implicated in insulin resistance, glucose metabolism changes, and DM. The pathways underlying such alterations are not always know but an in *vitro* essay with PIs and NRTIs showed altered adipocyte functions and decreased adiponectin, a positive regulator of insulin sensitivity, due to an increased expression and secretion of pro-inflammatory cytokines [65]. In another study, the PI indinavir has been implicated in inducing insulin resistance by acutely blocking transport of glucose by the insulin-sensitive glucose transporter GLUT4, a mechanism not found in non-HIV patients with DM [62].

Dyslipidemia in HIV population can result from both uncontrolled HIV disease and clinical restoration after HAART initiation. Individual, demographic and genetic traits besides the specific side effects of the antiretroviral combination contribute greatly to the type and degree of dyslipidemia seen in this population [66]. According to the D:A:D, a consortium assessing adverse events of anti-HIV drugs, the risk associated to certain PI’s (indinavir, lopinavir/ritonavir, abacavir) was consistently lower than the one calculated to the annual increment in risk associated to advanced age and current smoking habit [67]. The use of lopinavir/ritonavir [68], stavudine [63], efavirenz [69] and nelfinavir, zidovudine/lamivudine and didanosine/stavudine [70] have already been reported as causative of dyslipedemia by at least one of the following mechanisms (i) increased TG levels, (ii) increased LDL-c levels, and (iii) increased HDL-c levels.

Aside these specific cardiovascular risks, CVD have been reported as adverse effect with some ARV drugs, independently of metabolic disorders [71,72]. A meta-analysis indicated an increased risk of myocardial infarction in patients exposed to abacavir (RR 1.92, 95% CI 1.51-2.42), and an increased risk associated with each additional year of exposure to indinavir (RR 1.11, 95% CI 1.05-1.17) and lopinavir (RR 1.22, 95% CI 1.01-1.47) [72]. A prospective observational study from the D:A:D consortium showed that combination antiretroviral therapy was independently associated with a relative increase 0f 1.26 times in the rate of myocardial infarction *per* year of exposure during the first four to six years of use [58]. Despite these minor metabolic unbalances described for many antiretroviral drugs, it is important to keep in mind that the morbidity and mortality risks for HIV patients not on HAART are much higher than the risks seen with any antiretroviral drug or combination of drugs.

### Targeting MetS risk factors in the HIV population

As aforementioned, the success of HAART implementation allowed HIV individuals to live longer and as a consequence they can accumulate the same cardiovascular risks exposure described for the general population, such as high blood pressure, DM, dyslipidemia, and smoking habit, which is known to be a more prevalent risk in the HIV infected than in general population [73]. A French nationwide cohort of HIV^+^ adults on HAART showed that nearly half the patients were overweight or obese at HAART initiation and 20% of the patients gained excessive weight within 2 years of HAART initiation [58]. Additionally, a retrospective cross-sectional study reported that, even though less common than among the general population, obesity and overweight were more prevalent in comparison to wasting in the HIV population [74].

In a recent cohort of HIV-infected patients receiving care in HIV clinics in US, 38.2% of patients were in either the moderately high-risk or high-risk categories, of whom 77.9% were current or past smokers, 74.2% had high blood pressure, 71.5% had elevated baseline LDL-c levels, 70.5% had low HDL-c levels, and 35.8% had MetS [75].

A recent study has suggested an exclusive map for estimating the correct risk of cardiovascular disease in these patients that should include, besides traditional risk factors, specific factors for HIV patients like viral factors, immune activation, chronic inflammation and side effects of antiretroviral therapy [75].

In managing hyperlipidemia, the decision to use lipid-lowering therapy or to switch antiretroviral therapy regimens should be individualized. Unfortunately, at least one-fifth of HIV-infected outpatients with high cardiovascular risk who were eligible for pharmacologic treatment did not received recommended interventions and so treatment goals could not be achieved [75]. Effective management of dyslipidemia in HIV individuals is essential to reduce cardiovascular risk but presents multiple pitfalls due to interactions between HAART and lipid-lowering drugs [76]. A primary effort in treating dyslipidemia in HIV patients is selecting lipid-lowering agents capable to work effectively to restore lipid metabolism while maintaining proper interactions with HAART [77].

Beyond their canonical activity in inhibiting HMG-CoA reductase, statins might attenuate inflammation associate to the low-level viremia which can lead to a higher risk of age-associated non-AIDS morbidity and mortality. In fact, there is evidence showing that patients who maintained virologic suppression on effective HAART obtained additional survival benefit from the use of a statin [77].

As for the general population, cigarette smoking is thought to be the most important cardiovascular risk factor among HIV patients. More than half of the subjects evaluated in a cross-sectional analysis were current or past cigarette smokers, and smoking conferred a 2.35 (95% CI = 1.92-2.87) risk for myocardial infarction [78]. Cessation of smoking was more likely to reduce cardiovascular risk than either the choice of the ART regimen or the use of lipid-lowering drugs [79]. When assessing carotid intima-media thickness as a surrogate marker of CVD in HIV cohort, only gender, age, BMI, hypertension and family history of CVD remained as significant factors associated with thickened carotid intima-media, suggesting that traditional CVD risk factors may play a major role in CVD among HIV population [57].

HIV individuals submitted to short-term exercise training can undergo reductions in the waist-to-hip ratio and in the amount of visceral fat, and reductions in the levels of cholesterol, triglyceride, and LDL-c and such changes may improve some of the adverse metabolic effects associated to HAART [80]. The assessing of aerobic training in improving abdominal fat accumulation and metabolic disorders in HIV infection revealed that it reduced visceral fat, lipid disorders, basal blood lactate and coronary heart disease and may be particularly important for patients with marked dyslipidemia [81].

## Conclusions

The current HAART-modified natural history of HIV infection has practically turned the disease into a manageable chronic condition. As such, special attention should be paid to both imperfect control of HIV replication and long-term adverse events linked to drugs used in the therapeutic scheme. Even though the benefits of HAART use are overwhelmingly greater than possible MetS and CVD risks, close management of those patients is called for, especially due to the fact that general population risk factors now overlap with specific ones in this population, even though the former are usually more prominent than the latter.

Thus, MetS in HIV populations ought to be closely monitored and controlled by programmatic and comprehensive public measures. These findings call for an integrated management strategy, including smoking cessation policies, diet modification, and regular physical activity planning. Finally, comprehensive educational measures are needed and further research is instrumental to assess the barriers to implement preconized interventions and to achieving recommended treatment goals that are singular to the HIV-population.

## Abbreviations

AHA: American heart association; BMI: Body mass index; CVD: Cardiovascular disease; D:A:D: Data collection on adverse events of anti-HIV drugs; EGIR: European group for study of insulin resistance; HDL-c: High density lipoprotein cholesterol; hsCRP: High sensitivity C-reactive protein; IFN-α: Interferon- α; IL-6: Interleukin-6; IDF: International diabetes federation; LDL-c: Low density lipoprotein cholesterol; MetS: Metabolic syndrome; NCEP/ATP III: National cholesterol education program/adult treatment panel III; NCEP/ATP III: National heart, lung and blood institute; NNRTI: Non-nucleoside reverse transcriptase inhibitors; NRTI: Nucleoside reverse transcriptase inhibitors; PI: Protease inhibitors; SMART: Strategies for management of anti-retroviral therapy; TG: Triglycerides; TNF-α: Tumor necrosis factor; DM: Type 2 diabetes mellitus; VEGF: Vascular endothelial growth factor; VLDL-c: Very low density lipoprotein cholesterol.

## Competing interests

The authors declare that they have no competing interest.

## Authors’ contributions

AAP: drafted the manuscript; MCNF: drafted the manuscript; AGP: conceived and drafted the manuscript. All authors read and approved the final manuscript.
